# Medical Organization

**Published:** 1875-08

**Authors:** A. A. Lyon

**Affiliations:** Shreveport, La.


### MEDICAL ORGANIZATION.

BY A. A. LYON, M.D., SHREVEPORT, LA.

This is an age of organization, an evidence, it would seem, of progress and expansion. Almost every department of business has its organization. Our Boards of Trade represent the commerce; then we have spinner's, workingmen's, press and pharmaceutical associations, etc. The mighty agricultural interest is represented by the Grange, and the more exalted ecclesiastical by its councils, assemblies, conferences, synods, etc., etc.

The advantages of this sort of co-operation are manifest and multiform, and with the continued rapid increase of our population and territorial extent, and consequent wider separation of distant geographical points, they become more and more apparent.

Not less in the business of medicine than in others, and in some respects much more is it important to avail ourselves of the benefits that flow from this species of combination, and the brief observations that follow are designed to encourage, as far as possible, the formation everywhere throughout the country of medical societies.

We are justly proud of our American Medical Association. Still, as at present constituted, it does not represent the profession of the United States. The same is also true within the bounds of many, if not all, of our State societies. How is this? The State societies are too often mere collections of physicians without regard to their connection with minor local societies, and in some instances even their standing as practitioners. From these imperfectly constituted bodies are chosen delegates to the central association, and, as it not unfrequently happens, these delegates are anybody that wants to go, and feels able to pay his way, regardless of his ability properly to represent the profession of his State. This, then, is not true representation. How shall we get it? By the universal establishment, as just suggested, of medical organizations—local medical societies, which, though in themselves distinct, shall act in harmony with, and be, in a certain sense, subordinate to similar associations above. In other words, let every town, village or country neighhood, in every county in all the States, that can enlist and bring to the front as many as six or more M.D.'s, in good professional standing, have its regularly organized medical society.

Now, assuming that such societies existed everywhere, meeting as frequently as varying circumstances would permit, and sending annually their chosen representatives to the State Society, which in turn and in like manner shall be represented in the National Association, we will have that perfectly systematized organization that the necessities of the profession demand.

While we advocate further improvement, yet we would not be understood as unduly depreciating our medical associations. We recognize the fact that they have already done great good, and are still continuing in the same direction. The rapid advances within the past few years, especially in the Southern States, in the matter of medical organization, is really an astonishment, and augurs well for the future.

Alabama, Mississippi and Arkansas are especially noticeable for the interest they have taken and the progress they have made, while nearly all of the other States publish handsome and well gotten-up volumes of annual transactions.

But let me push still further ahead: For our encouragement we may note, that to medical associations the profession and the world are indebted for the universal recognition and adoption, by all respectable physicians, of that grand code of medical ethics, that in its purity and the fullness of its principles and love, ranks second only to the Bible, and forms an unerring guide in the adjustment of all professional differences that are liable to arise, either among physicians themselves, or between the physician and patient. Through our societies, too, the profession has grown in general esteem, and added to its strength and dignity, inasmuch as the public has learned more of us, and, perhaps, more fully appreciates our claims; and now, when a society exists, the physician who holds himself aloof feels it necessary to offer an apologetic explanation why he seems thus deficient in professional spirit, for the people demand it of him. Moreover, it is through the agency of such associations that we can most efficiently battle with charlatanism and quackery that, like festering sores, attach themselves to our professional body, working damage.

We hope further, too, by this means, to effect a reform where one is sadly needed, viz.: in the matter of medical education. The annual delivery by our multitudinous competing schools of thousands, more or less, of graduated physicians (?) after a few months attendance (we might say nominal attendance) upon a course of medical lectures, is a grievous wrong, both to the profession and the public. The ignorance in the profession that necessarily results from this condition of things is calculated, in our judgment, more to mar its dignity, corrupt its purity, create distrust in its genuineness, and to foster and encourage quackery, in its many forms, than all things else combined.

We are almost prepared to say, with a medical friend, that the licensing power should be transferred from the colleges to the various State associations. "Let the colleges and hospitals teach medicine," says he, "but let the physicians in each State decide who shall practice in their respective bounds." The suggestion is a good one, provided we can get our State associations up to the propestandard. Whether it be practicable or not is the question. Yet, if we may not attain to this point of control, we certainly may hope to put ourselves in position to check, in some measure, what may properly be styled a most shameful abuse.

But independent of the advantages of medical organization in the generic, as set forth above, by which the profession of medicine is magnified and strengthened in its relation to the other learned professions and the general public, there are others of a more personal and specific character that cannot but commend themselves to the true physician.

In the first place, ours is a calling designated to ameliorate, as much as possible, human suffering, and as conscientious men, it should be our daily endeavor, more and more, to advance ourselves in the knowledge of its principles and the most approved modes of their application; and while it is, doubtless, true that most of us take and read our medical periodicals, still there are advantages in personal interchange of thought that are not reached by merely reading the pages of a journal, while participation in the literary exercises of a society coerces, as it were, improvement in some measure.

Again, "in a multitude of counsellors there is safety," and the frequent coming together of a body of intelligent physicians for the purpose of discussing the great truths of their profession, of exchanging opinions and clinical experiences, cannot but be beneficial to every doctor present, and thus indirectly to his patients. The true physician does not recognize the quackish principle of the secret nostrum vendor "of keeping to himself that he may enrich himself," but stands ever ready, through the proper channels, and in the proper way, to afford the benefit of his knowledge to the profession, and through the profession to the world's suffering sick wherever found.

Still further, meeting in this sort of socio-medical style, as illustrated in our smaller societies, is productive of advantage, in that it personally acquaints one physician with another, and not unfrequently results in the establishment of warm friendships, when previously existed but a formal professional acquaintance. It is a noticeable fact that in most of our municipal communities, especially the smaller ones, where medical societies do not exist, there is often a want of that personal cordiality that is implied in the professed fraternity we claim. Certain it is, that the tendency of local medical societies is to break down that disposition to hypersensitiveness and jealousy that not unfrequently arises in the professional intercourse of physicians, all of which tends, in greater or less degree, to diminish our power and influence, and serves, in some measure, as a bar to that usefulness that we should all aspire to.

We are aware that we have advanced no new thought in what has been above written, neither are we unmindful of the fact that it is much easier to talk and write about how things should be done than it is to do them, and while it may not be practicable to carry out, in full, all the plan suggested. We, nevertheless, feel assured that a great deal more can be done than has yet been, and a conclusion reached through personal observation and experience of the beneficial results of medical organization. If, therefore, these thoughts, thus rudely expressed, may be instrumental in the establishment of even one active little town or county society, we shall feel that we have not written in vain.

